# Adaptive Neural Network Structure Optimization Algorithm Based on Dynamic Nodes

**DOI:** 10.3390/cimb44020056

**Published:** 2022-02-07

**Authors:** Miao Wang, Xu Yang, Yunchong Qian, Yunlin Lei, Jian Cai, Ziyi Huan, Xialv Lin, Hao Dong

**Affiliations:** 1 School of Computer Science and Technology, Beijing Institute of Technology, Beijing 100081, China; 3220201093@bit.edu.cn (M.W.); 3120205507@bit.edu.cn (Y.Q.); 3120201035@bit.edu.cn (Y.L.); 3120201001@bit.edu.cn (J.C.); 3220200891@bit.edu.cn (Z.H.); 3220201066@bit.edu.cn (X.L.); 2 Suzhou Automotive Research Institute, Tsinghua University, Suzhou 215299, China; donghao@tsari.tsinghua.edu.cn

**Keywords:** Adaptive Neural Network Structure, genetic algorithm, Hebb’s rule, Pearson correlation coefficient

## Abstract

Large-scale artificial neural networks have many redundant structures, making the network fall into the issue of local optimization and extended training time. Moreover, existing neural network topology optimization algorithms have the disadvantage of many calculations and complex network structure modeling. We propose a Dynamic Node-based neural network Structure optimization algorithm (DNS) to handle these issues. DNS consists of two steps: the generation step and the pruning step. In the generation step, the network generates hidden layers layer by layer until accuracy reaches the threshold. Then, the network uses a pruning algorithm based on Hebb’s rule or Pearson’s correlation for adaptation in the pruning step. In addition, we combine genetic algorithm to optimize DNS (GA-DNS). Experimental results show that compared with traditional neural network topology optimization algorithms, GA-DNS can generate neural networks with higher construction efficiency, lower structure complexity, and higher classification accuracy.

## 1. Introduction

Nowadays, artificial neural networks scale rapidly as performance increases. However, traditional artificial neural networks use full connections between layers, which leads to redundant structures that waste hardware and software resources. And the existing network structure optimization algorithm has a complicated construction process and high time complexity, which is not conducive to application.

So, in this paper, we aim to: (1) enhance the adaptability of the neural network construction, with pruning method to remove redundant structures in the network; (2) ensure the functionality after network structure simplification, with the help of heuristic methods.

Inspired by the in-generation constructing process of biological neural networks, we presented an adaptive neural network algorithm based on dynamic nodes. In order to remove redundant network structures and make the network neurons adaptive, we need to establish a way to measure the correlation between neurons. Firstly, we consider using Hebb’s rule [1] to quantify the correlation. Hebb’s rule is one of the basic synaptic plasticity rules in the brain. When the neurons on both sides of a connection are activated simultaneously, this connection should be retained; otherwise, it should be deleted [2]. However, it requires that the neurons on both sides of the connection be activated simultaneously. In practice, we found that this condition is harsh. Sometimes, even the two neurons are not activated, they still have a certain correlation (this will be proved in the experimental part). Thus, we also used the Pearson correlation coefficient [3] to measure the correlation of neurons in adjacent layers and designed our Dynamic Node-based neural network Structure optimization algorithm (DNS). There are two steps in DNS, namely the generation step and the pruning step. In the generation step, the network generates hidden layers. In the pruning step, the network uses Hebb’s rule or Pearson’s correlation to measure the importance of connections and prune according to this. In DNS, both the number of neurons in the network and the structure of synapses are dynamically changed, and both adapt to the current task.

Inspired by the among-generation construction process of biological neural networks, we combine genetic algorithm (GA) [4,5] to optimize our DNS algorithm. When used to solve complex combinatorial optimization problems, GA can usually obtain better optimization results with faster speed [6]. It has strong versatility and robustness and is suitable for parallel computing [7]. So, in our GA-DNS algorithm, the neural network model generated by DNS is used as the seed network of GA, and the final result of optimal neural network model is generated by GA.

In summary, our main contributions are:

(1) Inspired by the construction process of the biological brain, we propose a Dynamic Node-based neural network Structure optimization algorithm (DNS) to remove redundancy in neural network structure; (2) Explore the feasibility of using GA to optimize DNS.

Through experimental comparison, we prove that DNS can significantly simplify the network structure, and has high modeling efficiency. In addition, when combined with GA, the GA-DNS could largely enhance the accuracy and availability of DNA, even maintain a good performance compared to other SOTA methods.

The structure of this paper is: Section 2 describes the related work of network topology optimization, and introduce the design methods and flowcharts of DNS and GA-DNS algorithms. Section 3 designs three experiments: GA-DNS performance comparison experiment, pruning algorithm selection experiment, and pruning threshold experiment. Finally, the discussion and conclusion is elaborated in Section 4.

## 2. Materials and Methods

### 2.1. Related Works

This work attempts to solve the issue of artificial neural network topology optimization. There are two main ways to solve this problem: (1) generate a complex network structure and then remove the redundancy through the pruning algorithm [8]; (2) evolutionary neural network [9].

The method of generating a neural network first and then pruning is to start with a small-scale network structure and continuously generate hidden layer neurons in an iterative process until the network’s performance reaches the expectation. It is a bottom-up design idea. Kwok and Yeung classified the construction algorithms [10], among which the classic methods are Dynamic node creation(DNC) algorithm [11] and cascade correlation algorithm(CC) [12]. The idea of DNC construction is simple, and only one sigmoid node is added to the network at a time [11]. Therefore, DNC has a high time complexity when the network scale is too large, making DNC difficult to apply. So, CC was born. The construction process of the CC method is similar to that of the DNC, but the BP algorithm is replaced by a correlation-based training method during training, which makes the learning speed of the CC algorithm fast. Sin et al. proposed a module construction idea [13] to solve construction of CC is slow. Sin added a network module instead of one neuron. The module includes multiple neurons or a new hidden layer. Mezard went one step further and proposed the Tiling algorithm [14]. Tiling can add hidden layers and units in hidden layers at will until convergence. In 2018, Kamath proposed EnvelopeNets to build neural networks [15], which allows CC not to be limited to static expansion methods. It can be seen that the current CC learning efficiency is high, but the accuracy is slightly insufficient. GA-DNS has computational efficiency close to CC while maintaining high classification accuracy.

After the network becomes complex, the pruning method needs to remove redundancy. Reed classified pruning algorithms in 1993 [16], and now the more commonly used method is the pruning method based on correlation. In 2015, Han S proposed a new compressing neural networks based on this pruning method [17]. Then, he also proposed a new pruning algorithm, DSD [18], which prunes the branches based on the importance of the connection between the front and back layers [19]. Frankle’s paper in 2018 pointed out the importance of sparse structure to the network [20], and it makes exploring better pruning algorithms a hot research topic. In 2019, Denttmers proposed a new pruning idea to find the best connection scheme given the sparseness of the network, that is, sparse momentum pruning algorithm [21]. In the same year, Lin and Liebenwein’s work pointed out that it can be pruned by generating adversarial networks [22] or by sampling [23]. In 2020, Tang combined the pruning process with cybernetics to reduce noise interference during the pruning process and allow the pruning to be performed in a near-stable system [24]. The pruning method of DNS belongs to the correlation-based pruning method. Unlike Tang et al., this pruning method realizes pruning from the perspective of bionics. An Evolution Algorithm (EA) [25] is a random search algorithm that can simulate natural selection and evolutionary processes. NEAT is considered a typical EA, which realizes network structure and weights co-evolution. NEAT [26] is a method of optimizing the network topology. It directly encodes the weight and structure through mutation and lossless recombination. HyperNEAT [27] is a variant of NEAT, and the difference is that HyperNEAT chooses indirect encoding, called Compositional Pattern Producing Networks(CPPN) [28]. CPPN allows the repetition and symmetry of the neural network structure, which more accurately reflects the composition of the human brain. Subsequently, biological inspiration took NEAT one step further. In 2010, the ES-HyperNEAT [29] method was inspired by biological coding, allowing denser bases in the coding of CPNNs. At the same time, ES-HyperNEAT uses a quadtree structure to determine the density and location of hidden nodes. In 2019, CoDeepNEAT [30] used EA and distributed to improve the performance of NEAT. However, NEAT and its optimization algorithms have shortcomings. The computational complexity of NEAT is very high. The computational complexity of GA-DNS is lower than that of NEAT. AmoebaNet-A [31] give evidence that evolution can obtain results faster with the same hardware, especially at the earlier stages of the search, and it surpasses hand-designs for the first time. Xue et al. introduce a novel EA-NAS algorithm based on a multi-objective modeling of the network design problem to design accurate CNNs with a small structure [32]. The use of reinforcement learning is also a design direction of neural structure evolutionary algorithms [33]. In neural structure optimization algorithms without evolutionary algorithms, DARTS [34] is based on the continuous relaxation of the architecture representation, allowing efficient search of the architecture using gradient descent. Wang R et al. also proposed an improved DARTS algorithm [35]. At the same time, a sequential model-based optimization (SMBO) strategy can also be used to optimize the neural network structure [36]. Mingxing Tan et al. proposed a neural architecture search algorithm—EfficientNet to uniformly scales all dimensions of depth/width/resolution using a simple yet highly effective compound coefficient [37].

### 2.2. Dynamic Node-Based Neural Network Structure Optimization Algorithm

The construction process of a biological neural network is complex to simple [38]. In the initial state, biological neurons are connected. In the subsequent learning process, some of the connections of neurons will begin to “weaken” or “enhance” with the frequency of signal transmission between neurons. This construction process is heuristically applied to the construction of neural networks, and an adaptive neural network algorithm based on correlation analysis–neural network structure optimization algorithm based on dynamic nodes(DNS) is proposed. DNS is a heuristic algorithm that can complete the construction and pruning operations of the network structure simultaneously in the iterative process and realize the optimization of the network structure.

**Figure 1:**
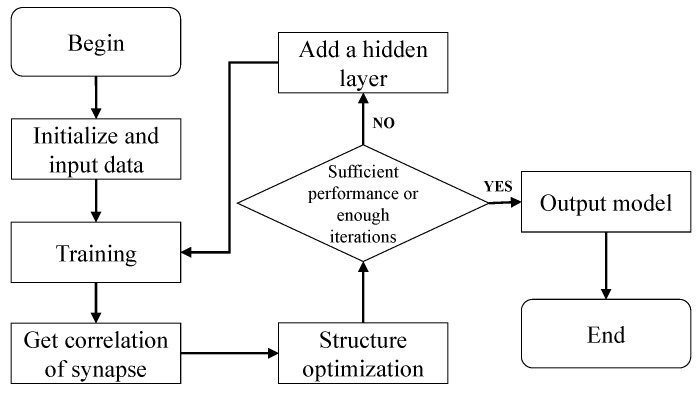
DNS algorithm.

The network only contains a fully connected input layer and an output layer in the initial stage. After one or several rounds of output are completed, the connections between neurons and neurons in the neural network structure are pruned according to the pruning strategy. The pruning strategy reduces the structural complexity of the model while ensuring specific performance. After pruning, if the stopping condition is not reached, a layer of hidden neurons is added to continue the iteration and pruning operations until the network performance meets the set requirements or reaches the set maximum number of iterations. Unlike cascade correlation networks, DNS will add neurons layer by layer, and the newly added hidden layer is located between the previous layer of neurons and the output layer. It is fully connected to other hidden, input, and output layers. In the initial state, neurons in the same layer are not interconnected. DNS algorithm is a process similar to brain growth and development. The data generated in the iterative process determines whether the connection in the model is deleted or retained. In summary, the DNS algorithm is as Figure 1 shows.

DNS has a process of network complexity after the initial network input. The traditional network construction method adds neurons to the network one by one, which is inefficient. The complication of DNS is to add the entire layer of neurons directly and then optimize the structure through the pruning algorithm, which is more efficient. At the same time, the complexity of DNS has a bionic significance. It is a simulation of the construction process of a biological specific neural network [39] and a guarantee of network self-adaptation.

After the network becomes complex, the network uses the pruning strategy to prune the connections with lower correlation. The relevance of the connection can be understood as the scoring of the connection. The pruning algorithm based on Hebb’s rule and the pruning algorithm based on Pearson’s correlation coefficient is described below.

We divide the pruning algorithm involved into four categories to explain the differences of pruning methods with different principles, namely:

(1) Frequent pruning strategy based on Hebb’s rule.

The DNS network does pruning every *U* epochs. For the neurons on the both sides of connection C, each iteration gets M outputs, denoted as $C_{l},C_{r}$, subscripts *l* and *r* indicate left and right, *T* is neuron activation threshold, $T\in \left(0,1\right)$. The connection correlation γ is calculated as follows:

(1) $$f\left(C_{l}^{m},C_{r}^{m}\right)=\left\{\begin{array}{cc} 1 & C_{l}^{m}>T,C_{r}^{m}>T\\ 0 & other \end{array}\right.$$

(2) $$\gamma_{i}=\sum_{m=1}^{M}f(C_{l}^{m},C_{r}^{m})$$

(3) $$\gamma =\frac{\sum_{i=1}^{e\cdot U}\gamma_{i}}{e\cdot U}$$

In function (3), *e* means the number of iterations per epoch, *U* means the number of epochs; here, this is 5. The above three formulas illustrate the conditions of Hebb’s rule. Only when the neurons on both sides of the connection C are activated at the same time, the C is considered effective. $\gamma_{i}$ counts the number of samples in which the neurons on both sides of C are activated at the same time among the *M* samples participating in the iteration. Frequent pruning strategy based on Hebb’s rule is as follows:

(4) $$f\left(C\right)=\left\{\begin{array}{cc} 1 & \gamma \geq p\cdot M\\ 0 & \gamma <p\cdot M \end{array}\right.$$

In function, (4), *p* means pruning threshold, $p\in \left(0,1\right)$.

(2) Exponential weighted pruning strategy based on Hebb’s rule.

Method (1) Only focus on the information generated by the current iteration, and the training of the BP algorithm is a continuous process, so the exponential weighting method is used to combine information from previous rounds. The specific calculation process becomes:

(5) $$f^{u}\left(C_{l}^{m},C_{r}^{m}\right)=\sum_{j=1}^{e}f_{j}\left(C_{l}^{m},C_{r}^{m}\right)$$

(6) $$\gamma =\frac{\sum_{u=1}^{U}(q^{U-u}\cdot \sum_{m=1}^{M}f^{u}\left(C_{l}^{m},C_{r}^{m}\right))}{\sum_{u=1}^{U}q^{U-u}}$$

In function, Equation (6), *q* means the base of the exponential weighting method. Generally speaking, as the number of iterations increases, the effect of the network will get better and better.

**Figure 2:**
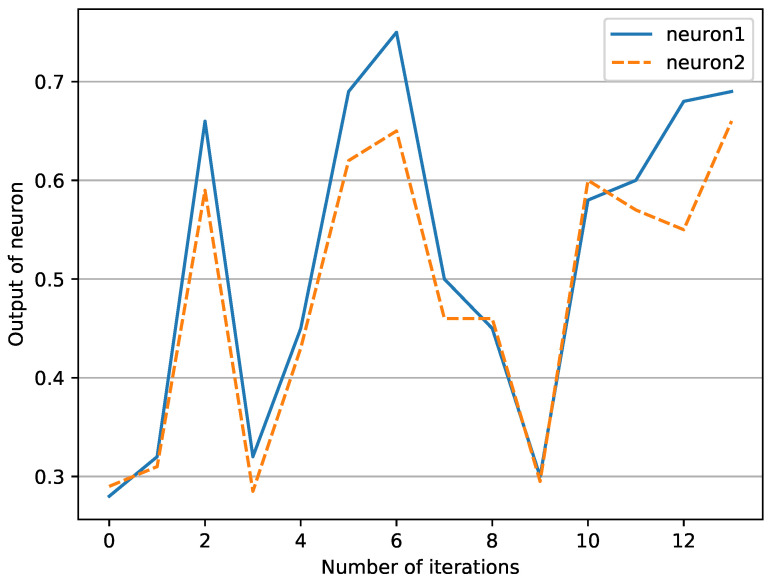
Neuron output curve.

However, the pruning strategy based on the Hebb rule may ignore some synaptic correlations. For example, when the output of the neurons on both sides of the connection does not reach the activation threshold, there may still be a certain correlation, but the Hebb rule will not calculate these correlations. As Figure 2 shows, the change rule of the neurons on both sides of the connection(namely neuron1, neuron2) has a sure consistency, and the correlation acquisition based on Hebb’s rule ignores this information. So, we also tried to use the pruning strategy based on the Pearson coefficient.

(3) Frequent pruning strategy based on Pearson correlation coefficient.

The design method differs from the previous only in the measurement of connection correlation. We use Pearson correlation coefficient here. The calculation of γ is:

(7) $$\gamma =|\mathrm{pearson}({\bar{C}}_{l},{\bar{C}}_{r})|$$

${\bar{C}}_{l}$ and ${\bar{C}}_{r}$ are the output vector groups obtained after e·U iterations. The pruning strategy is:

(8) $$f\left(C\right)=\left\{\begin{array}{cc} 1 & \gamma \geq p\\ 0 & \gamma <p \end{array}\right.$$

The range of the pruning threshold *p* is still (0,1). The larger the *p*, the harder the connection is to be retained. Unlike (1) and (2), *p* is not fixed.

(4) Exponential weighted pruning strategy based on Pearson correlation coefficient.

Similarly, the correlation γ is weighted exponentially.

(9) $$\gamma =\frac{\sum_{u=1}^{U}q^{U-u}\cdot \left|{\mathrm{pearsonn}}_{u}\left({\bar{C}}_{l},{\bar{C}}_{r}\right)\right|}{\sum_{u=1}^{U}q^{U-u}}$$

### 2.3. Optimize DNS with Genetic Algorithm

As previously described, the idea of DNS has specific feasibility. The main existing problems are as follows: (1) The final network generated by the pruning strategy based on Hebb’s rule or Pearson coefficient still has room for performance improvement. (2) The DNS network is not stable, and the algorithm can only control the overall optimization direction of the network. The final network structure may not be the same for the same data set and the same set of parameters. In order to solve the above shortcomings, this paper learned from the idea of an evolutionary neural network and combined DNS with GA. This combination can better solve the problem of network stability and improve the network’s generalization ability. Therefore, we proposed GA-DNS and designed a unique encoding method and genetic operator.

**Figure 3:**
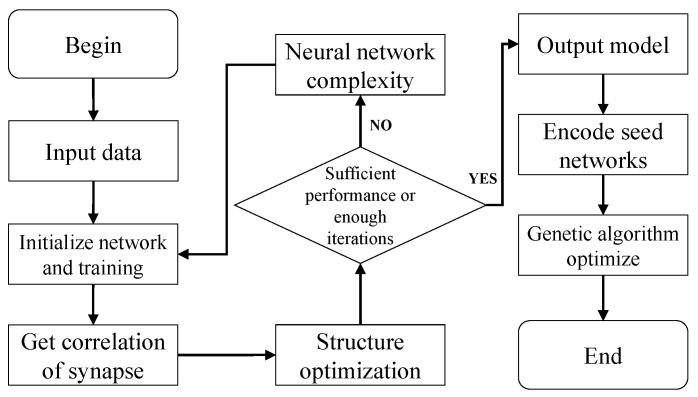
GA-DNS algorithm flowchart.

The flowchart of GA-DNS is shown in Figure 3. The neural network output by DNS is used as the seed network of GA, then we encode it by appropriate coding method, get locus of seed network and complete the population initialization process. Next, GA performs the iterative evolution of the population. When the maximum number of iterations is reached, or the maximum fitness has reached the threshold, the neural network with the highest adaptability can be obtained.

#### 2.3.1. Encoding Method

The research in this article only involves the connections between neurons, and there is no interconnection operation in a single neuron itself. In simple terms, there are two attributes of a connection. Attribute 1 is described as existence or non-existence, a binary classification value that identifies whether the connection exists in the existing model. Attribute 2 is described as the weight of the connection, which is a continuous float number. We can separately encode the connection status (existence or absence) and the connection weight of the connection and use the encoding method of two DNA strands to encode the structure and weight of the neural network generated by the DNS.

**Figure 4:**
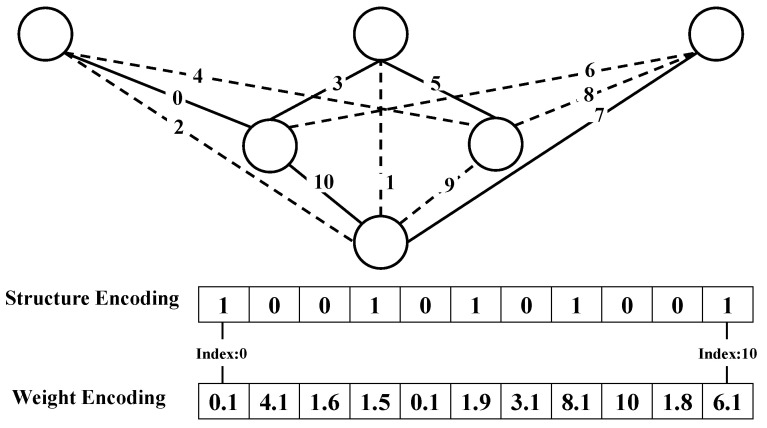
Schematic diagram of encoding method.

Figure 4 shows a simple diagram of DNS network coding. The upper part of the figure is a simple neural network structure diagram constructed by the DNS algorithm. The dotted line represents the connection deleted during the construction of the network. The solid line represents the real connection, which is the connection retained during the construction of the network. The connection between neurons is a simple binary value (exist or not), and the connection weight is a float number. The lower half of Figure 4 shows the network’s structural coding and weight coding. Structural encoding is the encoding of the connection state in the network, the value range of each locus is 0, 1, which is a typical symbol encoding method. The numbers 1 and 0, respectively, represent the existence and deletion of connections. The encoding length is the maximum number of connections network construction, including deleted connections and remaining connections. Weight encoding is an encoding method for encoding connection weights. Each gene of the weight encoding represents the connection weight of the connection corresponding to the index of the structure encoding. There is a one-to-one correspondence between structure encoding and weight encoding.

#### 2.3.2. Fitness Function

The DNS algorithm uses softmax loss function in the iterative process. Combined with the previous description, the reciprocal of the loss function can be selected as GA-DNS fitness function *F*:

(10) $$F=\left|\frac{1}{E}\right|$$

where *E* represents the loss function value of the model, here is softmax loss function. Generally speaking, fitness is a non-negative number, so the value of fitness *F* needs to add an absolute value.

#### 2.3.3. Selection Operator

The function of the selection operator is to select the parent individual. The parent individual is used to participate in genetic crossover and mutation operations. This paper intends to use the more commonly used roulette selection method and the optimal individual retention method as the selection operator. The advantages are: (1) ensure that the best individuals in the current population can be preserved intact to the next generation; (2) ensure that the better genes in the population (highly adaptive individuals) can be inherited to the next generation of individuals in the population. The specific process of selection operator is as follows:

1. Search for the individual with the highest fitness in the population and mark it as the best individuals.
2. Traverse the individuals in the current population. If the fitness of the current individual is higher than the optimal individual, replace the optimal individual.
3. Roulette selects the remaining next-generation individuals.
4. Iterative training of the current population generates the next-generation population, and the optimal individual does not participate in training.
5. The best individual enters the next generation and replaces the least adaptive individual in the offspring population.

#### 2.3.4. Crossover Operator

Structural coding is binary, so the crossover operator needs to be designed separately. The specific approch is:

1. Select parent, select two paired DNAs.
2. Parental crossover, crossover operation is performed on the two DNAs. The same gene remains unchanged for the part where the corresponding locus has the same gene, and the gene selection is performed for different genes.

It should be noted that step1 is selected by fitness value. Therefore, the calculation method of the degree of fit is as follows:

(11) $$\rho =\sum_{i=1}^{l}\left(f\left(x_{i}^{1},x_{i}^{2}\right)+\frac{1}{{\left(w_{i}^{1}-w_{i}^{2}\right)}^{2}}\right)$$

(12) $$f\left(x_{i}^{1},x_{i}^{2}\right)=\left\{\begin{array}{cc} 1 & x_{i}^{1}=x_{i}^{2}\\ 0 & x_{i}^{1}\neq x_{i}^{2} \end{array}\right.$$

Among them, ρ represents the degree of fitness between individuals. $x_{i}^{1}$, $x_{i}^{2}$ represent the coding information on the *i*-th locus in the structural coding of two individuals. $w_{i}^{1}$, $w_{i}^{2}$ represents the encoding information on the *i*-th locus in the weight encoding of the two individuals. *l* is length of genotype.

#### 2.3.5. Mutation Operator

The structural and weight coding methods are different, so the mutation operator design of structural coding and weight coding is different. The structural coding adopts the mutation method of basic bit mutation, and the weight coding adopts the mutation method of Gaussian mutation.

Structural coding adopts the mutation method of basic bit mutation because basic bit mutation is easy to implement and can ensure the diversity of the network structure to a certain extent, which is very suitable for the binary coding chain. The specific steps are as follows:

1. Parameter setting, the mutation probability of the basic position mutation pm.
2. Traverse gene locus, randomly generate a float number between 0 and 1, and traverse each gene in the individual structure code.
3. Replace gene. If the float number is less than the mutation probability pm, use the opposite character to replace the corresponding current code (when the locus is 1, replace it with 0, and vice versa); otherwise, it remains unchanged.

The weight coding adopts the mutation method of Gaussian mutation. The specific operation is as follows:

Suppose there is a population of individuals X, the genotype of X is expressed as $X=x_{1}x_{2}x_{3}\cdots x_{k}\cdots x_{l}$, the value range of the gene on the locus of X is $\left[U_{\min}^{k},U_{\max}^{k}\right]$. The Gaussian mutation operation process to $X^{\prime }=x_{1}x_{2}x_{3}\cdots x_{k}^{\prime }\cdots x_{l}$ is calculated as follows:

(13) $$\mu =\frac{U_{\min}^{k}+U_{\max}^{k}}{2}$$

(14) $$\sigma =\frac{2\cdot (U_{\max}^{k}-U_{\min}^{k})}{l}$$

The new gene $x_{k}^{\prime }$ is:

(15) $$x_{k}^{\prime }=\frac{U_{\min}^{k}+U_{\max}^{k}}{2}+\frac{2\cdot (U_{\max}^{k}-U_{\min}^{k})}{l}\cdot \left(\sum_{i=1}^{l}r_{i}-\frac{l}{2}\right)$$

## 3. Results

We design three experiments on the Nursery dataset from the UCI Machine Learning dataset. The first experiment compares the performance difference between GA-DNS and existing methods. The second experiment selects the best pruning strategy, and the third explores the pruning threshold’s effect on the pruning strategy.

The Nursery dataset comes from the UCI machine learning database. The total number of samples is 12,960, the number of features is 8, including five categories. The specific description of Nursery is as Table 1. Use one-hot encoding on the eight features in turn, and get a dataset with 12,960 samples, 27 features, and five categories. Each feature is a binary attribute. The ratio of cross-validated training set to test set is 8:2, and the order of the data in the sample is randomly shuffled.

The Adult Dataset is also a UCL machine learning database data set. It is extracted from the 1994 census database, including 48,842 samples, 14 attributes, and 81 attribute values. The classification task is whether the annual income exceeds 50,000. Firm-Teacher_Clave-Direction (referred to as FTCD) dataset also comes from UCL. It is a data set about music, including 10,800 samples and 16 attributes, quantifying a measure of 16th note music. The four categories correspond to the four-beat modes.

### 3.1. Comprehensive Performance of GA-DNS

This article first continues to use the Nursery dataset to explore the comprehensive performance of GA-DNS. The parameter settings of the GA part are shown in Table 2.

Accuracy (ACC), model structural coefficient (MSC), and total run time of the four GA-DNS, DNS, NEAT, and fully connected network (FC) models are tested simultaneously. MSC describes the sparseness of the final output neural network model. The larger the MSC, the more connections are deleted during the construction of the model, and the lower the structural complexity of the model. The calculation of total run time such as function (16). All experiments are done on a computer with a CPU model of i7-7700k, two GPU of NVIDIA GeForce GTX 1080, 32g RAM, and an operating system of Ubuntu 20.04.2. The experimental results are in Table 3.

(16) $$T_{run}=\frac{T_{R}}{T_{FC}},\left(T_{R}\in \left(T_{DNS},T_{GA-DNS},T_{NEAT},T_{FC}\right)\right)$$

In function (16), $T_{run}$ is “Total Run Time”, $T_{R}$ means the time it takes for the models to complete the classification.

It can be seen from Table 3 that GA has a very large impact on DNS, which greatly improves the accuracy of DNS on the Nursery data set. GA-DNS’s MSC is a slight decrease, which shows that the model structure is slightly more complicated than DNS, and the decrease also leads to a slight increase in Total Run Time. The GA-DNS’s accuracy is slightly flawed than the NEAT model, but it is better than the simple structure. NEAT is NeuroEvolution of Augmenting Topologies, a network of structural expansion and lacks a process to simplify the model. So, the MSC cannot be calculated for NEAT.

In order to ensure that the experiment is not affected by the dataset, the GA’s effect experiment on DNS has also been verified on Adult Dataset and FTCD Dataset. The experimental results are shown in Table 4.

The following points can be seen from the above experiments:

(1) The impact of GA on DNS is very large. The longer total run time is acceptable compared to the increase in accuracy. GA-DNS verifies that using GA to find the best network structure is feasible.
(2) Compared with the NEAT algorithm and fully connected network, the percentage of accuracy of the GA-DNS algorithm is slightly lower by about 4–6%. However, the advantage of the GA-DNS lies in a simpler network structure. Fully connected network is the most complex network structure, while NEAT is expanding the network structure. It is not as complicated as the BP network or as simple as the GA-DNS network structure.
(3) Both DNS and GA-DNS have a short Total Run Time. DNS is the fastest among them, but the cost is the lowest accuracy. GA-DNS has found a balance between accuracy and Total Run Time.

### 3.2. Reasonable Pruning Algorithm

The following is a comparative experiment on the Nursery data set to choose the best pruning strategy. The number of pruning is the same as the number of hidden layers. The basic parameters of each experiment remain the same, which are two hidden layers, and 10 neurons are added each time. The experimental results are in Table 5.

In Table 5, the accuracy, recall rate and MSC are selected as evaluation indicators. It can be seen that:

(1) The accuracy of the DNS network hovered between 70–80%, which shows that the neural network model optimized by the DNS algorithm has a certain ability to classify. However, the accuracy rate is not very high. Therefore, it can be further optimized.
(2) The model structure coefficient is between 0.55–0.75, indicating that the network scale has a certain degree of optimization compared with the traditional ANN, which is in line with the expected design of the algorithm, indicating the realization of the algorithm has certain feasibility.
(3) From the perspective of accuracy and model evaluation coefficients, compared with frequent pruning strategies, the pruning strategy based on exponential weighting is better.
(4) From the perspective of accuracy and model evaluation coefficients, the pruning strategy based on the Pearson coefficient is more effective than the pruning strategy based on Hebb’s rule.

### 3.3. Effect of Pruning Threshold

Different pruning thresholds *p* will lead to DNS generating different network structures, affecting the network’s performance and availability. Therefore, we adopt the method of controlling variables to observe the influence of different *p*. The DNS directly uses the exponentially weighted pruning strategy based on the Pearson coefficient. The parameter settings are as Table 6:

Among them, the number of hidden layers and the number of neurons added can be set based on experience, and there is no strict requirement. In this paper, based on the research theory of Frankle et al. [20], the number of additions is selected in the interval [$n_{in}$, $n_{out}$].

**Figure 5:**
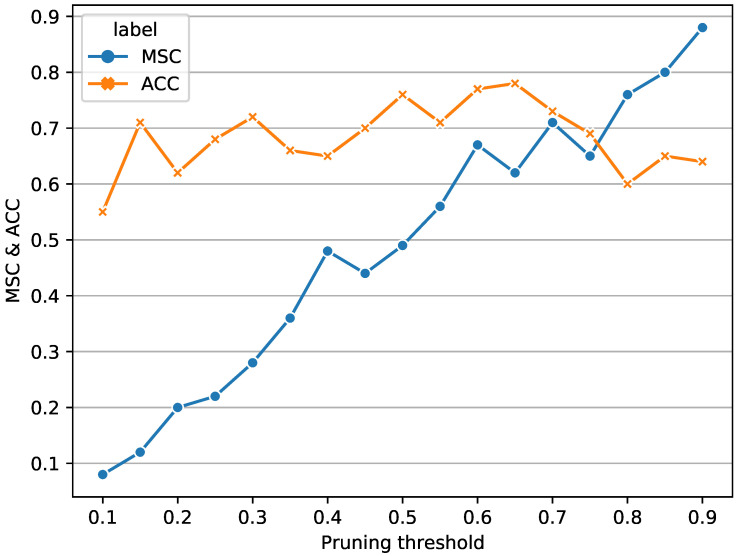
Line graph of the model accuracy rate and the model structure coefficient (MSC) under different *p*. The x-axis represents the pruning threshold and the y-axis represents the changes of MSC and accuracy.

Through many experiments, we found that when the accuracy of Δ*p* is 0.05, the change in the *p*-value caused the change in the model results was easily observable. Figure 5 shows the experimental results with p∈[0.1:0.05:0.9].

We can see from Figure 5:

(1) When the value of the pruning threshold *p* is small, the accuracy of the model is not high, and the model structure coefficient is very low. This means that the DNS algorithm retains most of the connections, the network structure is highly complex and over-fitting.
(2) As the pruning threshold *p* increases, the model structure coefficient increases simultaneously. This shows that some connections are pruned when the threshold *p* becomes larger and the streamlined network structure. However, the model’s accuracy fluctuates up and down, indicating that the model has not reached its optimal structure at this time, and there is room for further improvement in accuracy.
(3) When the pruning threshold is between [0.55, 0.7], the structure of the model has been greatly simplified, and the accuracy of the model is relatively stable at this time. However, the relationship between model structure coefficients and pruning threshold *p* is not strictly monotonic.
(4) When the pruning threshold is large enough, close to 0.9, the model structure coefficient is also close to the peak value, indicating that most of the connections in the model are deleted during the iteration process, and the accuracy of the model begins to decrease, indicating that the model is under-fitting at this time state.

The choice of pruning threshold *p* cannot be too wide or too small. The model with too small *p* value will enter the over-fitting state, and the model with too large *p* value will enter the under-fitting state, which is in line with the design philosophy of DNS. Different data sets may correspond to different optimal *p* values and network structures. On the other hand, the accuracy of the network is still not ideal, and the experimental results show that the accuracy is only 78% when the accuracy is the highest. So, the DNS algorithm needs to further optimize its performance. Simply adjusting the *p* will not bring further improvement inaccuracy. Therefore, it is necessary to use GA to optimize DNS to obtain a more available network.

In summary, GA-DNS is an algorithm with strong comprehensive performance and high modeling efficiency. However, GA-DNS is limited by the pruning threshold *p*, and practical applications require multiple experiments to determine the selection range of *p*, which limits the use of GA-DNS to a certain extent.

## 4. Discussion

Efficient structure, easy modeling, and strong usability are all essential contents in exploring neural network generation algorithms. This article first proposes a DNS method inspired by the construction process of biological neural networks. Then, we introduce GA-DNS to improve DNS’s accuracy and availability. Finally, the network generated by DNS is used as the seed network of GA, and the final output model is obtained after several population iterations. The overall performance of the network model output by GA-DNS on the Nursery dataset, Adult dataset, and Firm-Teacher_Clave-Direction dataset is reasonable compared to SOTA methods.

In our opinion, DNS represents the in-generation learning process of a biological neural network, while GA-DNS represents the among-generation evolution process of a biological neural network. GA-DNS shows higher accuracy than DNS, which means that the property of the population overall is stronger than an individual. We strongly believe that inspiration from biology will give us more hints towards better algorithms with better interpretability and higher learning efficiency.

The GA-DNS algorithm can be further optimized. Maybe we can optimize search methods to find the pruning threshold *p* and improve its engineering application capabilities in the future. It is also necessary to optimize the genetic algorithm to improve the accuracy of GA-DNS results. GA-DNS builds very quickly and guarantees a sure accuracy. In the future, we will consider using GA-DNS in the autonomous driving dataset to verify the practical application ability of GA-DNS.

## Figures and Tables

**Table 1:** Attribute information of Nursery.

Attribute	Detail
Parents	usual
	pretentious
	great_pret
Has_nurs	proper
	less_proper
	improper
	critical
	very_crit
Form	complete
	completed
	incomplete
	foster
Housing	convenient
	less_conv
	critical
Children	1
	2
	3
	more
Finance	convenient
	inconv
Social	non-prob
	slightly_prob
	problematic
Health	recommended
	priority
	not_recom

**Table 2:** Parameters of GA-DNS.

Parameter	Parameter Description	Value of Parameter
D	Maximum number of hidden layers added	2
V	Maximum number of hidden neurons added	10
$n_{in}$	Number of input neurons	27
$n_{out}$	Number of output neurons	5
U	Number of epochs between pruning	5
*p*	pruning threshold	0.65
population	Total number of individuals in the population	100
numIter	Population iterations	50
crossPro	Crossover probability of crossover operator	0.75
mulPro	Mutation probability of mutation operator	0.1

**Table 3:** Comparison of GA-DNS and DNS, NEAT, fully connected network, DARTS on Nursery.

Algorithm	Perfermance	MSC	Total Run Time
GA-DNS	89.62%	0.5667	0.92
DNS	79.41%	0.6841	0.89
NEAT	95.20%	–	1.10
DARTS	95.37%	–	1.14
FC	96.11%	0	1.00

**Table 4:** Experiment results of Adult Data Set and FTCD.

Dataset	Algorithm	Accuracy	MSC	Total Run Time
Adult	GA-DNS	91.81%	0.6075	0.91
	DNS	78.72%	0.6211	0.86
	NEAT	95.21%	-	1.04
	Hyper-NEAT	95.47%	-	1.07
	DARTS	96.81%	-	1.00
	FC	96.84%	0	1.00
FTCD	GA-DNS	92.23%	0.6450	0.88
	DNS	78.28%	0.6725	0.84
	NEAT	95.15%	-	1.10
	DARTS	96.02%	-	1.24
	FC	96.64%	0	1.00

**Table 5:** Experimental result on nursery.

	Purning Srategy Based on Hebb’s Rule		Purning Srategy Based on Pearson Coefficient	
	Frequent	Exponential Weighted	Frequent	Exponential Weighted
Performance	69.4%	75.2%	78.7%	83.5%
Recall rate	70%, 68%, 60%,	79%, 73%, 75%,	74%, 80%, 78%,	80%, 81%, 77%,
	62%, 71%	71%, 66%	72%, 62%	75%, 65%
MSC	0.56	0.72	0.57	0.68

**Table 6:** DNS algorithm parameter table of exponential weighted pruning strategy based on Person coefficient.

Parameter	Parameter Description
D	Number of hidden layers
V	Number of neurons added
M	Mini-batch subset sample number
$n_{in}$	Number of input neurons
$n_{out}$	Number of output neurons
U	Number of epochs between pruning
*p*	pruning threshold

## Data Availability

The data that support the findings of this study are openly available in: UCI Machine Learning Database at https://archive.ics.uci.edu/ml/datasets.php (accessed on 2 January 2022). The address of each data set is as follows: Nursery Data set: https://archive.ics.uci.edu/ml/datasets/Nursery (accessed on 2 January 2022); Adult Data set: https://archive.ics.uci.edu/ml/datasets/Adult (accessed on 2 January 2022); Firm-Teacher_Clave-Direction Data set: https://archive.ics.uci.edu/ml/datasets/Firm-Teacher_Clave-Direction_Classification (accessed on 2 January 2022).
